# Congenital Midline Cervical Cleft and W-Plasty: Our Experience

**DOI:** 10.1155/2018/5081540

**Published:** 2018-12-02

**Authors:** Abdullah Bahakim, Martine Francois, Thierry Van Den Abbeele

**Affiliations:** ^1^Department of Pediatric Otolaryngology, Robert Debre University Hospital, Paris, France; ^2^Department of Otolaryngology-Head & Neck Surgery, King Abdulaziz University, Jeddah, Saudi Arabia

## Abstract

**Objectives:**

Congenital midline cervical cleft (CMCC) is a very uncommon congenital anomaly of the midline anterior neck, and although it has very pathognomonic features (including nipple-like protuberance), it could be mistaken for other congenital neck lesions, such as thyroglossal duct cyst and branchial apparatus anomalies. Thus, it represents a challenging diagnosis. In this 21-patient series, we discuss the clinical features of CMCC, its pathophysiology characteristics, and its modalities management.

**Material and Methods:**

We conducted a retrospective chart review of children presenting with CMCC at our institution, between January 1998 and January 2016.

**Results:**

Twenty-one patients were identified with CMCC. Ages ranged between 1 day and 14 years. The length of the lesion varied from 0.5 to 5 cm, and the size of the skin tag varied from 0.2 to 1.5cm. No other significant associated anomalies were found. Surgery was the mainstay treatment, and no recurrence was found. W-plasty was used in most patients to close the defect.

**Conclusion:**

With a little more than 200 published cases, our series represents the largest series worldwide. The lesion is usually isolated, and no further investigation is required. Surgery is the mainstay of treatment, with complete excision being usually curative. It should be treated at an early age to prevent complications. In our experience, W-plasty was a good alternative to the most commonly used Z-plasty, in skin closure, with respect to both aesthetic and functional results.

## 1. Introduction

Congenital midline cervical cleft (CMCC) is a very uncommon congenital anomaly of the anterior neck that has very characteristic features: (1) a nipple-like protuberance (skin tag), (2) a skin defect in the middle with palpable subcutaneous fibrous cord, and (3) a blind sinus at the end. First described by Luschka in 1848 [1] and first reported in the English literature by Bailey in 1924 [2], it represents a variant (caudal extension) of cleft number 30 in Tessier's classification of craniofacial clefts [3]. The pathogenesis of CMCC is not well understood. Normally, the branchial arches grow medially in a cephalad to caudal direction with the first arch closing initially, followed by the others subsequently. Many theories have been proposed, including failure of fusion of the 1st and 2nd branchial arches in the midline during the third and fourth weeks of gestational age [4].

The standard procedure for CMCC closure is Z-plasty. However, there is some controversy over what procedure is best for CMCC. There have been a few case reports about W-plasty for CMCC, but no large case series presenting results on W-plasty for CMCC has ever been published. This is the first case series to present numerous cases on W-plasty for CMCC. In addition, to our knowledge, our study represents the largest case series of CMCC worldwide. Our purpose was to assess its clinical features, histopathology characteristics, and its modalities of management.

## 2. Materials and Methods

We conducted a retrospective chart review of patients with congenital midline cervical cleft at our institution, over 18 years, from January 1998 until January 2016. Our institutional review board approved this retrospective study and provided a waiver for informed consent.

**Figure 1:**
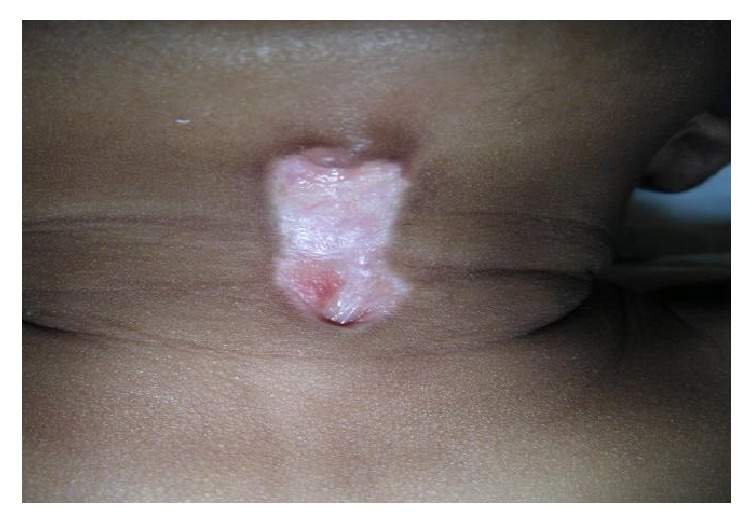
Clinical criteria of CMCC: superior nipple-like structure, inferior sinus, and atrophic skin in between.

Inclusion criteria for this study were as follows: (1) children presenting with CMCC who had its 3 typical features (Figure 1): cephalic nipple-like skin tag, caudal sinus tract, and midline atrophic skin in between; (2) only patients who underwent surgery for CMCC; and (3) each patient having a confirmed histopathology. Regarding the characteristics of CMCC, sometimes there was a cord-like fibrous band beneath the atrophic skin. In some cases, the skin tag was bigger than the fistula itself, while in other patients, purulent discharge due to infection was observed.

Exclusion criteria were as follows: (1) acquired cervical clefts and fistulas; (2) lateral located cervical lesions; and (3) patients not undergoing an operation.

Patients' charts, including history and physical examination, were thoroughly reviewed. The criteria that were analyzed included the following: age, sex, age at time of presentation, other associated anomalies, investigations, type of skin plasty used, histopathology reports, postoperative complications, and scar evolution. Complete lesion excision was done under general anesthesia. The lesion was closed by using W-plasty or Z-plasty or in a simple linear fashion.

## 3. Results

Patients' demographics were as follows. Twenty-one patients fulfilled the inclusion criteria (13 females and 8 males), with age of presentation ranging from 1 day after birth to 179 months (mean 21 +/-47 months, median 2 months). Most patients were operated on within a month after diagnosis, with age ranging from 2 to 181 months (mean 90 +/-291 months, median 12 months) (Tables 1 and 2.)

Regarding history and physical examination, all patients were born at term with normal birth weight. The length of the lesion varied from 0.5 to 5 cm, and the size of the skin tag varied from 0.2 to 1.5cm. Comprehensive head and neck examinations were performed. We did not find any associated abnormality, except for one patient (a 14-year-old girl) who presented with a limitation in neck extension due to a longstanding cleft. Concurrent congenital malformations were not present. The only anomaly found was for a boy with an undescended testis. No family history of CMCC was found.

Radiological tests were not routinely done. Ten patients had neck ultrasound and 2 patients had both neck ultrasound and MRI. Neck ultrasound showed a blind ending midline sinus tract arising from subcutaneous tissues, without any other anomalies. The thyroid was present in the normal position in all patients. MRI showed a lesion limited to the skin and subcutaneous tissues, 2-3cm in length and 0.5-1cm in width. The defect was a T1 hypointense, T2 hyperintense tract and with postcontrast peripheral enhancement. The boy with an undescended testis also had a renal ultrasound, which was normal, and was operated on later by a pediatric surgeon.

**Figure 2:**
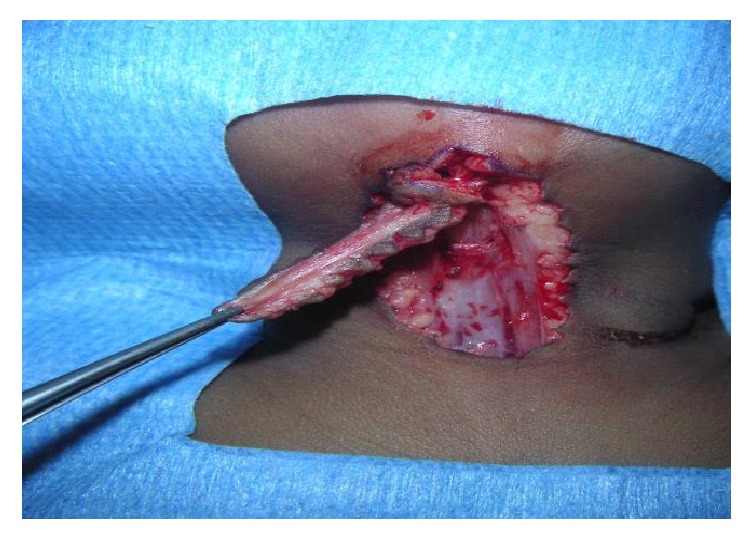
Perioperative view showing the excision of the CMCC.

**Figure 3:**
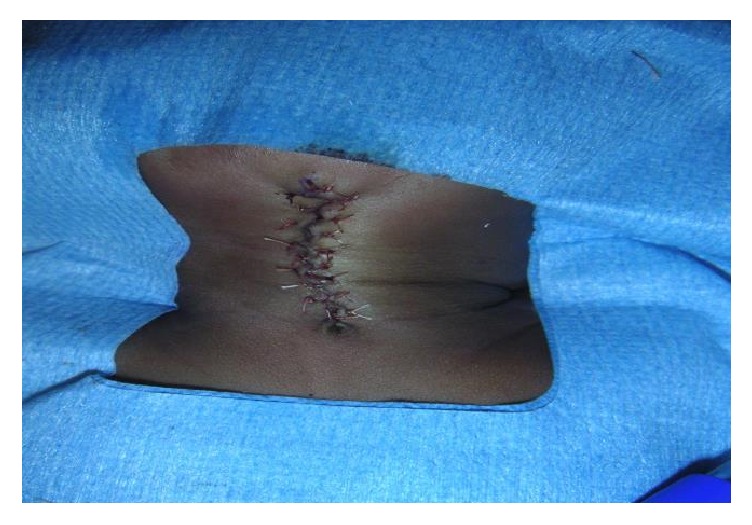
W-plasty after wound closure.

Regarding surgery, excision was done under general anesthesia. One surgeon performed 17 of the surgeries. Complete removal of the lesion included excision of the skin, subcutaneous tissues, and platysma muscle up to the level of the superficial (investing) layer of deep cervical fascia (Figure 2). Skin closure was obtained in a simple linear fashion in one patient, with multiple Z-plasty in 2 children and with W-plasty for the rest (18 patients, Figure 3). No drain was placed for any patient, as the lesion was superficial. Twelve patients were treated as a day care surgery and 9 patients were discharged on postoperative day one. Only oral analgesia was prescribed; paracetamol was usually sufficient. No child received antibiotics.

All patients were followed up at 7 days, one month, 6 months, and 12 months after the surgery. Twelve patients were followed up once a year for at least 5 years afterwards; 5 patients were followed up once a year for 3 years; and 4 patients were lost to follow up after their first annual visit (mean 45 +/-19 months, median 60 months) (see Table 2.)

One girl of African descent had a hypertrophic scar (from a linear closure). One boy of a Mediterranean origin had a small keloid in the scar (following W-plasty) that was managed successfully with 2 injections of 10 mg of triamcinolone acetonide. No wound reopening and no recurrence of the fibrous cord were noted in any patient. Neck movement was normal in all patients, without any contracture, except for the 14-year-old girl who presented initially with preoperative limitation of neck movement. This limitation gradually improved over 3 years after of the operation (see Table 3.)

Regarding histopathology, the lesions in each patient were composed of stratified squamous epithelium with parakeratosis covering bundles of striated muscles, without the normal skin appendages. The sinus tract for each patient was lined with pseudostratified columnar epithelium with seromucinous glands. A mild to moderate inflamed fibrovascular stroma with mononuclear inflammatory cells infiltrate was noted in 5 patients. In 4 patients, the skin tag was composed of normal skin with fibro-fatty tissues and occasional fibers of skeletal muscles. No associated cysts or thyroid tissues were noted in any patient.

## 4. Discussion

Congenital midline cervical cleft is a rare malformation of the midline anterior neck. It generally represents an incidence of 1.7-2% within the branchial arches malformations group [5], while some series report an incidence of less than 1% [6]. At our institution, we operated on 380 patients with a thyroglossal duct cyst, and only 21 patients had a CMCC over the same period of the study (18 years).

CMCC was first described by Luschka in 1848 [1], with the first report published by Bailey in 1924 [2]. The first full description of CMCC was presented by Ombredanne in 1949 [7]. Branchial apparatus development begins by the 4th week of gestation and is completed by week 6. It consists of 6 pairs of mesodermal arches lying in the transverse plane of the neck, numbered from the cranial to caudal direction. The 5th arch is small and regresses early. The rest of the arches are separated internally by endoderm-lined pouches and externally by ectoderm-lined clefts. Normally, they merge medially from the cephalad to caudal direction. In the case of CMCC, some mechanical factors or anomalies could result in disruption of this normal fusion. Multiple theories have been proposed to explain the pathogenesis of CMCC. The most accepted one is that the facial processes of the 1st and 2nd branchial arches fail to fuse during intrauterine life, due to either mechanical factors or vascular anomalies, which gives rise to ischemia and necrosis and results in a CMCC [5]. Knowing that 2nd arch anomalies represent 90-95% of all branchial anomalies, some authors have implied that the defect of fusion of the 2nd arch is the main cause of a cleft [8]. Gargan et al. [5] hypothesized that CMCC can fall into 2 main groups: (1) when there is a decreased cellular migration through the 2nd arch, an isolated CMCC could result; but (2) if the defect is in the 1st branchial arch, the results could be more severe, as with mandibular or tongue clefts. The extent of mesodermal proliferation within the cleft to close the fusion's gap determines the lesion severity, while improper interaction between the mesoderm and ectoderm could explain the absence of skin adnexal structures. Other theories include the following: rupture of a pathologic adhesion between the epithelium of the cardiohepatic fold and that of the ventral part of the 1st branchial arch [9], pressure from the pericardial roof on the developing branchial arches resulting in pressure necrosis and scarring [10], and failure of adequate mesenchymal ingrowth and amniotic adhesions [11].

Jakobson et al. [12] in 2012 did a genetic analysis on 3 cases of isolated CMCC. Two mutations were found: deletion of the pregnancy associated plasma protein A (PAPPA) and mutation in the SIX5 gene. His study concluded that these mutations do not directly cause the disease, but they can be contributing factors. Agag et al. [13] found that CMCC was associated with 13/14 de novo Robertsonian translocations.

The lesion is situated in the midline between the chin and suprasternal area, with variable length and width. Puscas [14] found a positive correlation between the size of the defect and the patient's age.

Several studies suggested that there is a predominance of CMCC in the Caucasian population, mainly in females, with a F:M ratio of 2:1 [15, 16]. However, Achard et al. in 2016 [17] found an equal ratio between males and females with CMCC, and Puscas [14] even found a male predominance, with 8 boys and 2 girls, in his published retrospective series in 2015. Consistent with most studies, we identified 13 females and 8 males with a CMCC (a ratio of 1.6:1), with most of them being Caucasian (Table 1). No previous published article has suggested any familial inheritance of a lesion.

Due to its characteristic clinical features, most cases of CMCC are noticed by a pediatrician during the initial neonatal examination and are then referred to an ENT surgeon soon after birth. On the other hand, some patients are referred later on in their life, as some families believe it is a simple birthmark, when the lesion does not disappear on its own as the child grows up, or when there is a seromucinous discharge.

Early intervention is required to correct this anomaly. If left untreated, it can result in neck webbing and contractures (as in the case of the 14-year-old girl in our study) and mandibular deformities, such as micrognathia and exostosis-like protuberances [18].

As shown in several studies and case reports (Van Der Staak et al. [19], Sinopidis et al. [20]), we believe that CMCC is mostly an isolated problem. Only one patient had another associated anomaly (a boy with an undescended testis). In their retrospective case series of 8 patients, Achard et al. [17] found only one patient with an atrophic kidney. Some authors found that CMCC can be associated with other anomalies like thyroglossal duct cyst [21], bronchogenic cyst [22], or a midline cleft from mandible to sternum [23].

Several methods have been proposed for defect closure after excision including a simple vertical closure, Z-plasty, and W-plasty. A simple linear closure can be done if the defect is small [24], but it might lead to contracture formation and limitation in neck movement, according to Gargan [5] and Gardner et al. [9].

A better way of dealing with the defect in most cases is to use a single or multiple W-plasty or Z-plasty, especially in case of a large lesion. They are better in terms of cosmetic results and subsequent functional results and have less risk of cicatricial contracture formation. However, Z-plasty can lead to hypertrophic scarring in the oblique limbs [16] along with triangle tips depression and necrosis if the angles are too acute (<30 degrees).

**Figure 4:**
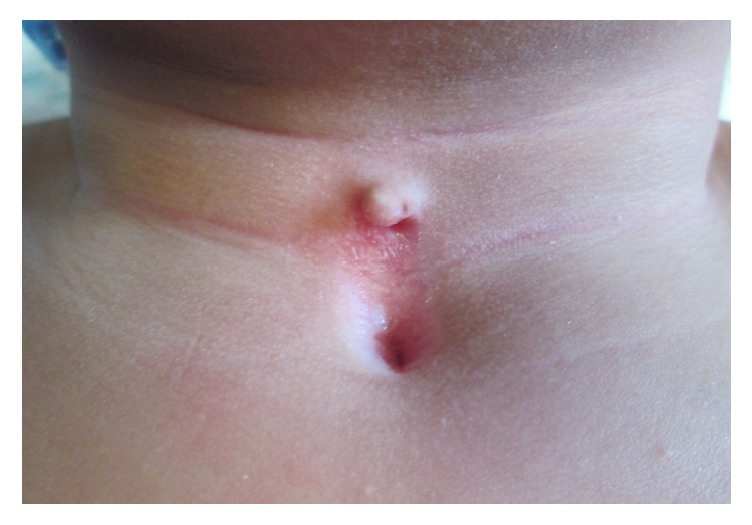
CMCC in a 2-year-old boy.

**Figure 5:**
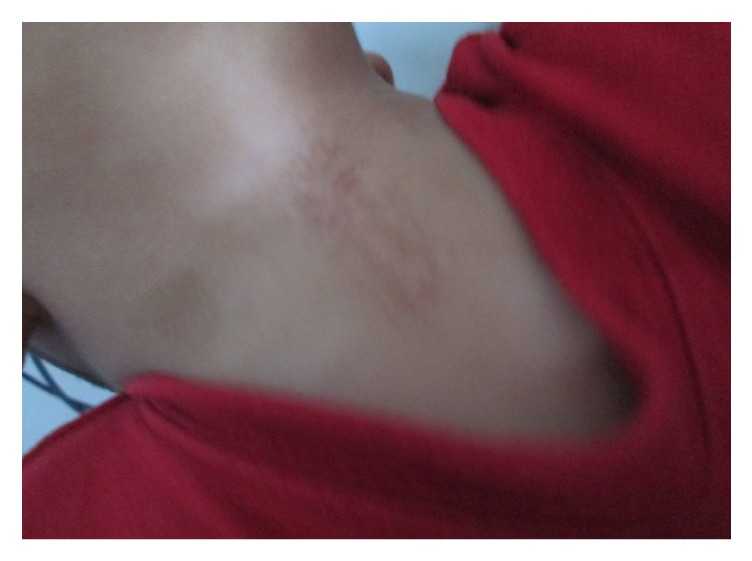
Postoperative result in the same patient at 12 months.

**Figure 6:**
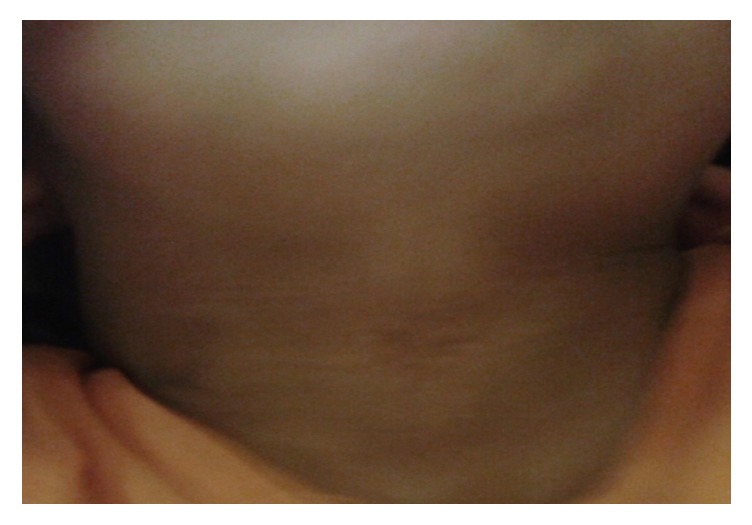
18-month postoperative result in the same patient in Figure 4.

Although in the literature Z-plasty is the most commonly used way of incision closure in CMCC [17, 23, 24], we mostly used W-plasty (in 18 patients). In our experience, we found that it gave long-term satisfactory cosmetic and functional results. Preoperative and postoperative views at 12 and 18 months can be seen in Figures 4, 5, and 6, respectively, of a 2-year-old boy who has been treated with W-plasty, showing a good esthetic result. It has some advantages over Z-plasty: for example, segments with shorter limbs are used and it does not cause an overall lengthening of the incision. Designing the W-plasty before incision can be a bit tricky and confusing. The surgeon has to be careful when drawing the small intertwining triangular lines around the lesion, and he must follow the drawing precisely when doing the incision, so the two sides will interpose perfectly after excising the lesion. The apices should measure at least 60 degrees (ideally between 60 and 90 degrees) and are placed at a distance of 5 mm from each other and 3-5 mm from the scar. Triangle limbs should be 3-5 mm long (maximum 6 mm) and the end portions should be in an acute angle (<30 degrees) to avoid a dog ear effect [25, 26].

The classic histopathology findings included the following: (1) a skin tag: stratified squamous epithelium with occasionally striated muscles or cartilage; (2) a main lesion: stratified squamous epithelium with surface parakeratosis and absence of dermal adnexal structures, with mild inflammatory infiltrate of lymphocytes, plasma cells, and neutrophils possibly present; and (3) a sinus tract: squamous or pseudostratified columnar epithelium. A respiratory epithelium, thyroglossal dust cyst, or its remnants were reported in some cases [27].

## 5. Conclusion

CMCC is a very rare entity among the congenital branchial arc anomalies. It is usually an isolated lesion, and diagnosis is confirmed with a clinical examination by an expert otolaryngologist. It does not require further evaluation or investigation, if no other lesion is suspected. CMCC needs to be treated early to prevent complications, such as neck contractures or mandibular growth defects. Complete surgical excision is indispensable. In our experience, W-plasty provides satisfactory functional and esthetic results, with no recurrence encountered if properly excised.

## Figures and Tables

**Table 1:** Ethnicity and sex of patients.

**Ethnicity**	**Number of patients**	**Sex**
**Caucasian **	14	9 females
		5 males

**Mediterranean**	4	2 females
		2 males

**Asian**	2	1 female
		1 male

**African**	1	1 female

**Table 2:** Measured demographic variables.

	**Mean** **∗**	**Median** **∗**	**Standard deviation** **∗**
**Age at presentation**	21	2	47

**Age at surgery**	90	12	291

**Follow up**	45	60	19

*∗*Values in months.

**Table 3:** Closure types.

**Method of Closure**	**Number of patients**	**Complications**
Linear	1	1 (Hypertrophic scar)

Z-plasty	2	None

W-plasty	18	1 (Keloid)

## Data Availability

The data used to support the findings of this study are available from the corresponding author upon request. The corresponding author will verify first with the institutional review board before supplying the data, in order to protect the patient's privacy.
